# A comparison of Kneipp hydrotherapy with conventional physiotherapy in the treatment of osteoarthritis of the hip or knee: protocol of a prospective randomised controlled clinical trial

**DOI:** 10.1186/1471-2474-10-104

**Published:** 2009-08-19

**Authors:** Martin Schencking, Adriane Otto, Tobias Deutsch, Hagen Sandholzer

**Affiliations:** 1 Kneipp Clinic (Sebastianeum & Kneippianum) Bad Wörishofen, Kneippstrasse 08, D-86825 Bad Wörishofen, Germany; 2 Dept of Primary Care, University of Leipzig Medical School, Ph.-Rosenthal-Strasse 55, D-04103 Leipzig, Germany

## Abstract

**Background:**

The increasing age of the population, especially in the western world, means that the prevalence of osteoarthritis is also increasing, with corresponding socioeconomic consequences. Although there is no curative intervention at present, in accordance with US and European guidelines, pharmacotherapeutic and non-pharmacological approaches aim at pain control and the reduction of functional restriction.

It has been established that hydrotherapy for osteoarthritis of the hip or knee joint using serial cold and warm water stimulation not only improves the range of movement but also reduces pain significantly and increases quality of life over a period of up to three months. Weight reduction is important for patients with osteoarthritis of the hip or knee. In addition, conventional physiotherapy and exercise therapy have both been shown, at a high level of evidence, to be cost-effective and to have long-term benefits for pain relief, movement in the affected joint, and patient quality of life.

**Methods/design:**

The study design consists of a prospective randomised controlled three-armed clinical trial, which will be carried out at a specialist clinic for integrative medicine, to investigate the clinical effects of hydrotherapy on osteoarthritis of the knee or hip joint, in comparison with conventional physiotherapy.

One hundred and eighty patients diagnosed with osteoarthritis of hip or knee will be randomly assigned to one of three intervention groups: hydrotherapy, physiotherapy, and both physiotherapy and hydrotherapy of the affected joint. In the first group, patients will receive Kneipp hydrotherapy daily, with water applied in the form of alternate cold and warm thigh affusions (alternating cold and warm water stimulation is particularly relevant to the knee and hip regions).

Patients in the second group will receive physiotherapy of the hip or knee joint three times a week. Patients in the physiotherapy-hydrotherapy combination group will receive both joint-specific physiotherapy three times a week and alternate cold and warm thigh affusions every day. Follow-up assessments will be on three levels: clinical assessment by the investigator; subjective patient assessment consisting of a patient diary, and questionnaires on admission and at the end of the treatment phase; and a final telephone assessment by the external evaluation centre. Assessments will be made at baseline, after two weeks of inpatient treatment, and finally after a further ten weeks of follow-up. The primary outcome measure will be pain intensity of the affected joint in the course of inpatient treatment, judged by the patient and the investigator. Secondary outcomes include health-related quality of life and joint-specific pain and mobility in the course of the study. Statistical analysis of the results will be on an intention-to-treat basis.

**Conclusion:**

This study methodology has been conceived according to the standards of the CONSORT recommendations. The results will contribute to establishing hydrotherapy as a non-invasive, non-interventional, reasonably priced, therapeutic option with few side effects, in the concomitant treatment of osteoarthritis of the hip or knee.

**Trial Registration:**

***Trial registration number***: NCT 00950326

## Background

Osteoarthritis (OA) is the most common and most enduring physical impairment of patients in the western world; age is known to be one of the most important risk factors for this disease. It affects approximately 10% of all people over 60, with estimated socioeconomic consequential costs of about 60 billion dollars per year in the US alone [1].

As well as considering osteoarthritis to be a result of mechanical irritation, attrition and erosion of the cartilage matrix, another scientific hypothesis postulates a loss of vital cartilage cells and cartilage matrix degeneration [2]. Obesity, with a BMI >26, is considered a predictive marker for osteoarthritis of the knee or hip joint [3].

The clinical picture of osteoarthritis is characterised mainly by joint pain, crepitus, joint stiffness after rest, hyperthermia, and progressively restricted movement. Along with overweight, preventable or at least modifiable risk factors are physical activity, nutrition, and hormonal influences [4].

Although there is no curative intervention at present, in accordance with US and European guidelines, pharmacotherapeutic and non-pharmacological approaches aim to control pain and reduce reduction of functional restriction. Non-pharmacological therapy comprises a multidisciplinary approach consisting, for example, of instructions for weight loss and exercise therapy; pharmacotherapy comprises non-opioid analgesics, non-steroidal anti-inflammatory drugs (NSAIDs) including COX-2 inhibitors, topical analgesics (e.g. capsaicin cream), and opioid analgesics, as well as intra-articular steroid and hyaluronic acid injections [5].

Various epidemiological studies showed that NSAIDs were prescribed for 86.9% of patients, analgesics for 29.9% received, chondroprotectives for 7.6%, and gastroprotective drugs for 36.9% [6]. In just one year in France, 17 million prescriptions and five million days' absence from work were documented because of osteoarthritis [7].

NSAIDs have gastrointestinal side-effects, so complementary and alternative medicine (CAM) is increasingly offering concomitant therapeutic options. The prevalence of the use of CAM in osteoarthritis is estimated to be 40%: vitamin supplements, celery extract, fish oil, and garlic extract are often part of patient self-medication [8]. CAM further offers evaluated therapeutic procedures, including acupuncture, various herbal agents such as stinging nettle, boswellic acid, devil's claw extract, and proteins such as glucosamine [9,10]. Besides acupuncture and glucosamine, systematic reviews have also evaluated pool therapy, balneotherapy, thermotherapy, and exercise therapy, and found beneficial outcomes for pain and movement restriction in osteoarthritis of the hip or knee [11-14].

Patient surveys showed that 80% of patients with osteoarthritis had used several complementary medical procedures in the previous month; 52% used over the counter medicines. Other approaches taken were dietary measures (71.5%); mind-body techniques (42.4%), salves and ointments and other topical applications (38.1%), taking vitamins and/or trace elements (32.9%), herbal preparations, and various physical manipulation techniques (21.4%) [15].

Several basic articles describe Kneipp hydrotherapy (e.g. in the form of alternate cold and warm thigh affusions) as having an active effect on the vessels by causing initial local vasoconstriction followed by reflexive vasodilatation, which activates the cutaneous circulation [16-18]. Furthermore, modulation of hormone production was scientifically demonstrated for cortisol and adrenaline [19-21].

Immunologically, hydrotherapy increases serum concentrations of alpha-2 macroglobulin, IgM antibodies, and complement factor C3 [22]. In addition, using alternate cold and warm back affusions, positive effects were found on immunoregulation, in the sense of an increase in resistance and facilitated activation of cell-mediated (Th1) immune reactions, as measured by the mediating cytokines IFN-gamma and IL-2 [23].

Serial cold and warm water stimulation hydrotherapy for osteoarthritis of the hip or knee joint showed improvement of restricted joint mobility, along with significant pain reduction and increase in quality of life over a period of up to three months [24,25].

**Figure 1:**
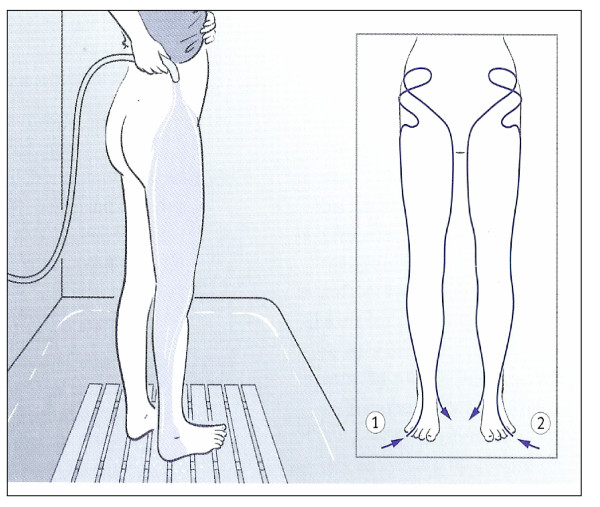
**Alternate thigh affusion according to Kneipp – flow direction (repeated cold/warm water stimulation)**. The thigh affusion is poured starting from the back of the foot up the outer aspect of the back of the leg, slowly over the buttocks ("starting away from the heart going towards the heart"), from there moving to the front aspect of the leg, lingering in the groin, and finally pouring the water over the inner aspect of the leg downwards.

In particular, serial application of alternate cold and warm thigh affusions (Figure 1) has established itself as concomitant therapy for osteoarthritis of the hip or knee; this therapeutic application is also indicated for adjuvant treatment of peripheral arterial disease and insomnia. The thigh affusion is poured starting from the back of the foot up the outer aspect of the back of the leg, slowly over the buttocks ("starting away from the heart going towards the heart"), from there moving to the front aspect of the leg, lingering in the groin, and finally pouring the water over the inner aspect of the leg downwards [26].

Weight reduction is important for patients with osteoarthritis of the hip or knee. In addition, conventional physiotherapy and movement therapy have both been shown, at a high level of evidence, to be cost-effective and to have long-term benefits for pain, movement in the affected joint, and patient quality of life [27-29].

This randomised clinical trial will be funded by the Otto Schönfisch Foundation, Bad Wörishofen, Germany (Project number: Dr.Wa/zie/2.7.08).

## Methods/design

### Research aims and questions

The main aim of this three-armed clinical study will be to determine the effects of hydrotherapy with alternate cold and warm affusions of the thigh in the concomitant treatment of osteoarthritis of the hip or knee. The main outcome measures are pain reduction, improvement in the quality of life, and increased movement in the affected joint.

### Research questions

1. Does Kneipp hydrotherapy show a measurable effect in the sense of altered clinical findings in the affected knee or hip joint?

2. How effectively does hydrotherapy influence the clinical parameters of pain, range of movement, and function of the affected joint?

3. How effectively does hydrotherapy affect the secondary outcome measures of quality of life, pain experienced, mood, and blood pressure?

4. Do hydrotherapeutic applications simply provide short-term therapeutic success or are there long-term changes in the clinical findings?

5. Are the clinical effects of hydrotherapy comparable with those of conventional physiotherapy of the affected joint?

6. Are the clinical effects of hydrotherapy used as monotherapy comparable with a combination of hydrotherapy and physiotherapy of the affected joint?

7. Do unwanted effects or side effects occur with hydrotherapy?

### Study duration

January 2009 – December 2010

### Design

This study was designed as a prospective randomised controlled clinical trial with three arms, which is to be carried out at a German clinic specialised in integrative medicine, to investigate the clinical effects of hydrotherapy on osteoarthritis of the knee or hip, as compared with conventional physiotherapy.

To achieve maximum scientific accuracy with respect to randomisation, random distribution, avoidance of selection bias, etc., the independent Department of General Medicine and Primary Care of the University of Leipzig Medical School will function as an external evaluation and testing centre.

The design and concept of this study, as well as its ethical validity, were reviewed and approved by the Ethics Committee of the Bavarian State Chamber of Physicians [Ethikkommission der Bayerischen Landesärztekammer], (Study Number 08032, dated 04.05.2008).

### Subjects

All patients of a German clinic for integrative medicine who have symptomatic osteoarthritis of the hip or knee joint, who meet the revised criteria of the American College of Rheumatology, who have agreed to participate in the study, given their informed consent and signed a data privacy policy statement, will be included in the study. Patients will be given an information sheet, which will tell them they will be randomly assigned to one of the three following treatment groups:

**In group 1**, patients will receive a specific hydrotherapeutic procedure in the form of alternate cold and warm thigh affusions (pouring on water) which will consist of repeated cold and warm water stimulation of the knee and hip region. Physiotherapy of other regions, such as the back, is permitted but there will be no specific physiotherapy of the hip or knee joint.

**In Group 2**, patients will be given physiotherapy of the hip or knee joint three times a week but no thigh affusions. Affusions of other regions, such as the back, are permitted.

**In Group 3**, patients will receive not only joint-specific physiotherapy of the hip or knee joint but also daily thigh affusions of alternate cold and warm water.

The patients will be informed of possible unwanted side-effects; in addition they will be told that they can withdraw from the study at any time without any fear that they will not continue to receive treatment.

The inclusion and exclusion criteria are to be found in Table 1.

**Table 1:** Eligibility criteria

**Inclusion criteria**	
1	Age ≥ 18 years
2	Symptomatic osteoarthritis of hip or knee (following the revised criteria of the American College of Rheumatology)
3	Willingness to comply with follow-up assessments and treatment
4	Ability to understand, read and speak German

**Exclusion criteria**	

1	Endoprothetic replacement of hip or knee joint
2	Inflammatory arthropathy of the hip or the knee
3	Acute, hot, red and swollen knee or hip joint (unknown focus)
4	Inflammatory system diseases which could interfere with the evaluation of the therapy procedure
5	CNS diseases, especially epilepsy
6	Anamnesis of deep vein thrombosis in the past 12 months
7	Severe lung disease such as e.g. COPD stages GOLD III – GOLD IV
8	Heart failure NYHA III – NYHA IV
9	Myocardial ischemia with or without intervention within the last 3 months before inpatient admission
10	Cancer in advanced stage
11	Large skin wounds or inflammatory and ulcerated dermatosis of the legs
12	Severe febrile infectious diseases Non treated hypertension
13	Participation in another clinical study within the past four weeks
14	Pregnancy

After the treating physician has clinically examined a patient with osteoarthritis of the hip or knee and confirmed that all the inclusion criteria and none of the exclusion criteria are met, the patient will be given the information sheet, a statement of informed consent, and a privacy policy statement for this clinical study. If the patient agrees to take part, the external evaluation centre will be informed, and will send the investigator a plain envelope with a randomisation number, treatment group assignment, and the necessary study documents.

### Randomisation and treatment allocation

Based on the results of a pilot study, a randomisation number list and treatment group assignment has been generated by software before the beginning of the main study. When the envelope is opened, the investigator and patient will come to know the treatment group assignment (group 1, 2, or 3) with a corresponding treatment plan in line with the study protocol, the patient diary for the inpatient treatment phase, and all the necessary assessment forms for both doctor and patient.

Prior to the study, all medical specialists in the clinic and attending therapists have been given a briefing on the study protocol, the ethical basis of this clinical study, the randomisation procedure, and the specific therapy for each treatment group, in line with the study protocol.

### Sample size

On the basis of the data from the pilot study, carried out between June 2008 and January 2009 at the Kneipp Clinic in Bad Wörishofen, Germany, on 30 patients with pre-existing osteoarthritis of the hip or knee, a sample size was estimated using G-Power 3.0.10 software (Faul F, Erdfelder E, Lang A.-G, and Buchner A, 2007). According to this calculation, a sample size of n = 180 patients will suffice to determine a possible interaction between the groups with respect to the primary outcome, with a statistical power of 95%.

### Interventions

**Figure 2:**
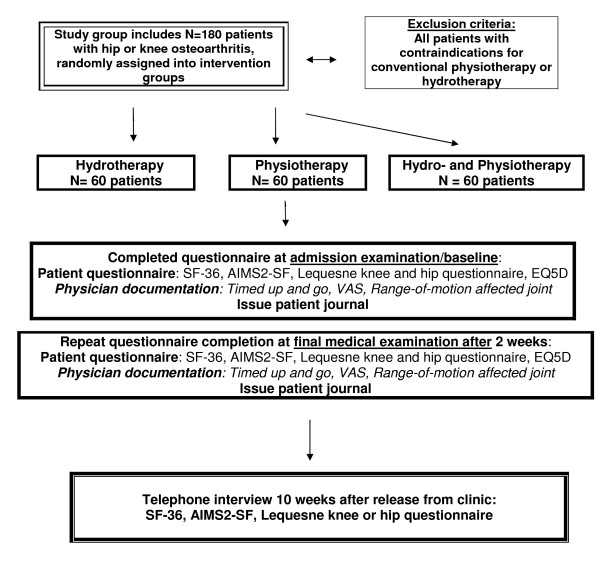
**Flowchart describing the participant flow through the study**. Diagramm over the participant flow through recruitment, randomization, baseline measurement, post-treatment and 3 month follow-up.

The study will divided into a **clinical or treatment phase **(two weeks' inpatient therapy) and a **follow-up phase **of a further ten weeks, ending with a telephone interview to be carried out by the external evaluation centre (Figure 2).

During the clinical phase the following interventions will be applied:

In **intervention group 1**, the patient will receive hydrotherapy appropriate for osteoarthritis of the hip or knee, in the form of daily thigh affusions of alternate cold and warm water. In this group, physiotherapy may also be given but not specifically for the affected hip or knee joint.

In **intervention group 2**, patients with osteoarthritis of the hip or knee will receive specific physiotherapy of the affected hip or knee joint three times a week, but **without **any disease-specific hydrotherapy. Because of the holistic approach of the clinic, however, these patients will still receive hydrotherapy at sites other than the affected joint, for example, alternate cold and warm affusions of the back or an ascending lumbar affusion.

In **group 3, the control group**, patients with active osteoarthritis of the hip or knee will receive specific, joint-related hydrotherapy in the form of a (daily) alternate cold and warm thigh affusions **as well as **joint-specific physiotherapy (three times a week).

### Diagnostic and biometric evaluation procedure

Each patient will undergo a medical examination at the time of admission and a final examination after two weeks of inpatient therapy, in which the general physical state and disease-specific previous medication and therapy will be documented. At the two medical examinations, pain intensity will be measured on a ten-point visual analogue scale, and 1) diagnostic assessment of the affected joint according to the neutral-zero method (hip joint: flexion and extension, internal and external rotation; knee joint: flexion and extension); 2) the timed "up and go" (TUG test) as an indirect assessment criterion of flexion and extension in the affected hip or knee joint; and 3) the body mass index (BMI) will be recorded in the case report form or physician's questionnaire.

**Independently of the doctor**, a standardised questionnaire will be filled out by the patient at the time of admission and after two weeks of inpatient therapy. These questionnaires will be sent to the evaluation centre directly after they are handed in.

The same assessment will be carried out in a telephone interview by the external study centre, ten weeks after the end of inpatient care.

The assessment includes A) the SF-36 test, B) the Lequesne hip or knee questionnaire, C) the AIM-2-SF Scales 2 Short Form, and D) the EQ-5D test.

**Figure 3:**
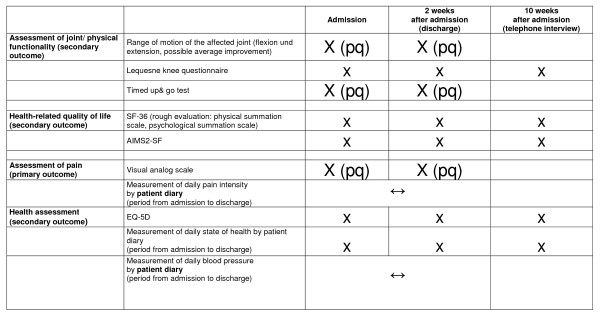
**Schedule of data acquisition**. [pq = physicians questionnaire]. EQ-5D = EuroQol questionnaire; AIMS2-SF = Arthritis Impact Measurement Scale.

In addition, each patient will be given a diary for self-reporting during inpatient treatment. This diary will be completed by the patient: it contains the subjective visual analogue scale, the current mental health state scale of the EQ-5 D test, and blood pressure measurements (filled out by the nursing staff) ((Figure 3)).

### Outcome measures

#### Primary outcome measure

The primary outcome measure will be the pain intensity of the affected joint during inpatient care, as assessed by the patient and the investigator. The patient rates subjective pain intensity in the patient diary on a ten-point visual analogue scale (0 = no pain; 10 = greatest pain imaginable); the doctor also records pain intensity around the affected joint on a ten-point visual analogue scale. The use, practicality, and validity of visual analogue scales have been sufficiently evaluated and are scientifically accepted [30,31]

### Secondary outcome measures

#### I) Mobility of the affected joint

The mobility of the affected hip or knee joint is documented by the investigator at the time of admission and the time of discharge from the clinic, using the neutral-zero method and range of motion scale. Flexion and extension, internal and external rotation are recorded for the hip joint, as are flexion and extension for the knee joint.

#### II) Quality of life rating for the patient with osteoarthritis, on the basis of the German version of the Arthritis Impact Measurement Scale (AIMS2)

The practicality, reliability and validity of the Arthritis Impact Measurement Scale (AIMS2) have been confirmed in many studies, and for this reason it is used in judging the quality of life in patients with osteoarthritis. Its inherent consistency and reliability are similar to those of the WOMAC score, with Cronbach's alpha at 0.77 or higher [32]. The patient will document this index at the beginning and end of the inhouse treatment period, and it will again be recorded by the external evaluation centre on the basis of the telephone interview at the end of the ten-week follow-up.

#### III) Joint-specific pain and function rating on the basis of the Lequesne index

The Lequesne index is a patient-centred assessment (self-rating procedure) in the form of a questionnaire. It is available in two closely related versions (hip or knee joint diseases), and includes pain, walking performance, and coping with daily living. The evaluation shows high reliability, validity, and sensitivity to change.

As a self-rating procedure, the Lequesne index is not subject to the influence of the investigator or therapist. There is no investigator bias, which always has to be recognised within procedures combining self-rating and rating by another person. The Lequesne index is therefore a suitable assessment tool for quality assurance [33,34]. The patient will document this index for the affected joint at the beginning and end of the inhouse treatment period, and it will again be recorded by the external evaluation centre on the basis of the telephone interview at the end of the ten-week follow-up.

#### IV) Rating the general health-related quality of life on the basis of the SF-36, short version

The SF-36 is an internationally recognised tool for ascertaining patients' health-related quality of life in a wide range of diseases. It comprises two summation scales and eight dimensions, which can be divided into the areas of "physical health" and "mental health". The inherent consistency (Cronbach's alpha) for "physical function" is between 0.77 and 0.93, between 0.73 and 0.85 for "pain", and between 0.57 and 0.75 for "general health perception". Its validity and reliability have likewise been evaluated scientifically [35,36]. The short version of the SF-36 that we use will be recorded by the patient at the beginning and the end of the inhouse treatment period and again by telephone by the external evaluation centre at the end of the ten week follow-up.

#### V) Rating the indirect flexion and extension ability of the hip or knee joint and general patient mobility through the timed "up and go" (TUG) test

In its original form, the TUG is an internationally recognised procedure for rating body balance and the danger of falling during an everyday task involving movement. For this reason, it is a standard procedure in geriatric and general practitioner assessment of individual patient mobility and risk of falling. The TUG's validity, inherent consistency, and reliability are scientifically proved [37]. To carry out the test, the patient sits on a chair with armrests, which is placed three metres from a wall. When prompted, the patient has to stand up, pause in front of the chair, walk the three metres to the wall, turn around without touching the wall, walk back to the chair, turn around again and sit down. The time required, use of aids, and general feasibility are measured. We use the TUG test, together with an estimate of general mobility, to rate flexion and extension of the affected knee or hip joint indirectly, as described previously in the scientific literature for the indication of osteoarthritis [38]. The TUG test will be administered by the investigator at the beginning of the study and again at the end of the inpatient treatment phase

#### VI) Standardised survey of state of health based on the EQ-5D test

The EQ-5D test is a standardised international procedure for self-rating of the patient's health status. It is divided into a general descriptive part and a pain-related part. The five dimensions rated in the descriptive part are mobility, self-sufficiency, daily activities, pain or discomfort, and anxiety or depression; the pain section of the EQ-5D test consists of the visual analogue scale in the self-rating procedure. In contrast to the SF 36 and the AIMS2, a more exact rating of everyday activities and their disease-related restriction is possible. The inherent consistency and validity of the EQ-5D test have been well evaluated scientifically [39].

In the EQ-5D self-rating procedure, results will be recorded by the patients at the beginning of the study and at the end of the treatment phase; the external study evaluation centre will also collect data by telephone ten weeks later.

#### VII) Blood pressure profile during the inpatient treatment phase

To demonstrate possible concomitant effects of the inpatient therapy on blood pressure – especially in the hydrotherapy group and the group combining hydrotherapy with physiotherapy – nursing staff will record the patient's blood pressure each day in the patient's diary.

#### VIII) Body weight changes during the inpatient phase, based on the BMI

The investigator will record the patient's BMI at the beginning of the study and at the end of the inpatient treatment phase.

### Statistical analysis

The data will be analysed using SPSS 15.0 (SPSS inc., 2006) software according to the intention-to-treat principle. Accordingly, all patients with valid values will be included in such a way that they remain in the groups in which they were originally randomised, irrespective of the treatment they actually received.

To check the homogeneity of the groups, a cross-sectional comparison of the baseline characteristics will be carried out. Various statistical procedures will be applied, depending on the scale level in each case. A factorial analysis of variance (one way ANOVA), if necessary with appropriate post hoc tests, will be carried out for the metric variables, body mass index (BMI), age and blood pressure, the scale values in SF-36, AIMS 2-F, visual analogue scale (VAS), and Lequesne index, the time required in the timed "up and go" test, and flexion and extension in the neutral-zero testing,. The nominal variables sex, affected joint (left, right, both sides) and the items of the EQ-5D will be analysed with chi-square tests. An analysis of variance with measured value repetition (ANOVA, repeated measures) will be performed for the longitudinal analysis of the primary outcome measure (VAS), and for the secondary outcome criteria (current mental health state, SF-36 sum scales, AIMS 2-SF scales, Lequesne index, time required in the timed "up and go" test, flexion and extension in the neutral-zero testing, and blood pressure) which are also metric variables. Should, contrary to expectations, there be any inhomogeneity between the groups with respect to important variables (baseline characteristics), adjustments will be made as far as possible in the scope of the longitudinal analysis. The secondary outcome measures on the nominal data level (EQ-5D) will be analysed with chi-square tests.

### Feasibility of the study design

A pilot study was carried out between June 2008 and January 2009 to check the practicability, the treatment protocols, and the randomisation procedure. Patients were recruited at a specialist clinic in Germany. A total of 30 patients with symptomatic osteoarthritis of the hip or knee joint were randomised into intervention group A (hydrotherapy), intervention group B (physiotherapy) or the control group C (physiotherapy and hydrotherapy). The results of this pilot study showed that all study procedures, therapeutic interventions, and randomisation procedures were practicable and feasible; there was no disqualification on account of unwanted adverse reactions or side effects. All physicians and therapists reported problem-free implementation of the study protocol and efficient study coordination.

## Discussion

The increasing age of the population, particularly in the western world, means that the prevalence of osteoarthritis of the hip and knee is also increasing, with corresponding socioeconomic consequences [40,1]. In the reasons for all visits to a general practitioner in Germany, osteoarthritis of the hip and knee come in places 14 and 34, respectively; they come in the third and ninth places for orthopaedic consultations [41].

Although there is currently no curative intervention, in accordance with US and European guidelines, pharmacotherapeutic and non-pharmacological approaches aim at pain control and the reduction of functional restriction. The development of more efficient, cost-effective, sustainable therapeutic strategies with fewer side effects is thus gaining in significance.

It has been established that hydrotherapy for osteoarthritis of the hip or knee joint using serial cold and warm water stimulation not only improves the range of movement in the affected joint but also provides significant pain relief and increases quality of life over a period of up to three months [11,25]. Even so, there is only scattered evidence from RCTs or meta-analyses which investigate the use of hydrotherapy for osteoarthritis with respect to its long-term and sustained effects [18].

For this reason, we designed this study to evaluate the efficiency of hydrotherapy of the affected joint, in the form of alternate cold and warm Kneipp affusions, in patients with symptomatic osteoarthritis of the hip or knee, in comparison with conventional physiotherapy.

The statistical analysis of the study result will follow the intention-to-treat principle.

The study has limitations, however: hydrotherapy in patients with symptomatic osteoarthritis will be studied in comparison with conventional physiotherapy in the context of a two-week inpatient stay in a well-known German specialist clinic for integrative medicine and centre for hydrotherapy, with ten weeks' subsequent observation. Possible secondary intervention parameters, such as simultaneous sports activity, movement therapy, nutritional therapy or dietary measures for weight reduction of obese patients with osteoarthritis during their inpatient stay were not included in the pilot study, nor will they be in the main study. For one thing, the hydrotherapy will not be continued during the ten week observation phase and, for another, the intake of phytotherapeutics and nutritional supplements will not be studied. These will be the subjects of further RCTs.

However, the results of our pilot study have shown that the study procedures, therapeutic interventions and randomisation procedure are all practicable and feasible. In addition we observed positive long-term effects of the hydrotherapy with respect to pain and quality of life.

This study design is based on the CONSORT statement for randomised, controlled studies [42].

## Competing interests

The authors declare that they have no competing interests.

## Authors' contributions

MS conceived the study, obtained the approval of the relevant ethics committee, organised funding for the study and wrote the manuscript. MS, AO and TD performed the data preparation and analysis. All authors have read the manuscript in its final version, have checked it, and provided critical comment.

## Pre-publication history

The pre-publication history for this paper can be accessed here:


